# Screening of Ready-to-Eat Meat Products for Hepatitis E Virus in Switzerland

**DOI:** 10.1007/s12560-018-9340-x

**Published:** 2018-02-28

**Authors:** Dominik Moor, Marianne Liniger, Andreas Baumgartner, Richard Felleisen

**Affiliations:** Risk Assessment Division, Federal Food Safety and Veterinary Office FSVO, Schwarzenburgstrasse 155, Bern, 3003 Switzerland

**Keywords:** Hepatitis E virus, Virus extraction, RT-qPCR, Raw meat sausage, Pork liver sausage, Risk-assessment

## Abstract

Seroprevalence data for pig herds suggested that there must be a relevant reservoir for hepatitis E virus (HEV) in Switzerland. To know more about the viral presence in ready-to-eat meat products, we screened pork liver sausages and raw meat sausages from the Swiss retail market for the presence of HEV. Testing was performed with a detection method where the virus extraction step was optimized. As for the performance of the improved method, the mean recovery rate for the mengovirus process control was 24.4%, whereas for HEV-inoculated sample matrices between 10.4 and 100% were achieved. The limit of detection was about 1.56 × 10^3^ and 1.56 × 10^2^ genome copies per gram for liver sausages and raw meat sausages, respectively. In the screening programme, HEV-RNA was detected in 10 of total 90 (11.1%) meat products, 7 of 37 (18.9%) liver sausages, and 3 of 53 (5.7%) raw meat sausages. Virus loads of up to 5.54 log_10_ HEV genome copies per gram were measured. All sequences retrieved from positive samples belonged to HEV genotype 3. The significance of the presented work was a current overview of the HEV prevalence in ready-to-eat meat products on the Swiss retail marked and an improvement of the extraction efficiency of the HEV detection method.

## Introduction

In Europe, the most important enteric viruses are hepatitis A virus (HAV), norovirus, enterovirus, rotavirus, and astrovirus (Le Guyader et al. 2000). Moreover, hepatitis E virus (HEV) increasingly received attention in recent past. This agent occurs worldwide with an estimated number of 20 million cases and 56,000 fatalities per year. Affected are mainly developing countries where hepatitis E is a waterborne infection and HEV genotypes 1 and 2 cause large outbreaks (Blasco-Perrin et al. 2016). In developed countries hepatitis E occurred relatively rarely and sporadically, but with a tendency of increase (SurvStat@RKI 2.0). It was assumed that the registered cases were mainly persons infected on trips to Asia or Africa (Federal Institute for Risk Assessment 2010). However, in recent years HEV was isolated from swine suggesting that hepatitis E is a zoonotic disease (Worm et al. 2002) and in Germany, it was shown that this zoonotic reservoir is responsible for autochthonous sporadic cases (Wichmann et al. 2008). Another investigation showed a rather high HEV-seroprevalence of 16.8% indicating that HEV is endemic in Germany (Faber et al. 2012). HEV-borne outbreaks, mainly due to contaminated water, are common in countries with poor hygienic conditions. Nevertheless outbreaks in developed countries are reported, but these were linked to the consumption of food containing pork liver. In France for example, a group of wedding participants got infected due to the consumption of an undercooked pork liver-based stuffing (Guillois et al. 2016) and in Australia, pork liver pâté served in a restaurant caused an outbreak (Yapa et al. 2016).

From the aforementioned six viral agents, only HAV has to be mandatorily reported in Switzerland. The recorded cases of hepatitis A are mainly due to travelling activities and the only food-borne outbreak with HAV was registered in the year 2000 where a shedder working in a bakery shop was identified as the source of infections (Schmid and Baumgartner 2012). In contrast to Germany, hepatitis E is currently not a reportable disease. Other than sporadic cases, outbreaks have to be reported, but to date such an incident was never registered in Switzerland. The lack of data from the official reporting system is a drawback but at least, a study with blood donors is available showing a low seroprevalence for HEV of 4.9% in Switzerland (Kaufmann et al. 2011). This prevalence was considerably below the values of similar investigations in England (13.5%), France (16.6%), and Denmark (20.6%). In contrast, a high prevalence of 60% for antibodies against HEV in pigs at slaughter was found (Wacheck et al. 2012). Another study carried out in the same period of time revealed a seroprevalence in domestic pigs of 58.1% and in wild boars of 12.5% (Burri et al. 2014). In a recent publication, two HEV isolates from a patient hospitalized in Switzerland with acute hepatitis and from a raw sausage containing pig liver were sequenced in full length. The analysis implied that the two isolates belong to the same virus strain and that they may form a Swiss-specific HEV genotype 3 subcluster (Kubacki et al. 2017). These studies suggested that there could be a relevant reservoir for HEV in Switzerland and it was therefore hypothesized by us that meat products from domestic pigs and wild boars are contaminated with HEV. To find out to what extent such contaminations might occur, domestic and imported meat products containing raw pork or pork liver were screened for the presence of HEV with a molecular detection method. Testing foods for the presence of viruses is time-consuming and laborious. A critical point is often the limited virus extraction efficiency of the methods. These difficulties result in the unsatisfactory situation that at least half of viral food-borne outbreaks are not recognized (Stals et al. 2012). We addressed this critical point to find a way to improve the sensitivity of a recognized detection method published earlier (Szabo et al. 2015). Finally, considerations about the risk of HEV-contaminated foods were made and possible consequences for authorities of food control and consumers were discussed.

## Materials and Methods

### Collection of Samples

A total of 90 ready-to-eat food products of domestic and imported pork liver sausages and raw meat sausages were purchased at retail shops in Switzerland between February and April 2016. Liver sausages should have passed a heating step. Local sausage specialties (“Mortadella cruda”, “Lebersalsiz”, “Salametti”) containing raw pork liver or raw game meat of deer, wild boar, chamois and ibex were provided by Agroscope, Research Division Food Microbial Systems. Samples were stored at 4 °C for short term or at − 20 °C for long term until virus extraction.

### Preparation of Food Samples for Virus Extraction

Extraction of virus particles from sample material was performed according to a published method (Szabo et al. 2015). The sample preparation step was slightly modified. For each food sample, a single extraction was performed. Samples of 2 g liver sausage or 5 g raw meat sausage were manually chopped using a surgical blade and transferred into a sterile 400-ml blender bag with side-filter (BagFilter^®^ P). Then 7 ml TRI Reagent^®^ Solution (Ambion), 5 ml sterile phosphate buffered saline (PBS, pH 7.4, Sigma) and 50 µl of a process control solution consisting of bacteriophage MS2 (DSM 13767) and mengovirus (MeV) strain vMC_0_ (ATCC VR-1597) diluted in PBS were added. The amount of both process control viruses added to each sample corresponded to about 10^5^ and 10^6^ genome copies for the screening study of ready-to-eat meat products and the method optimization experiments, respectively. Samples were homogenized for 2 min at highest velocity using a Stomacher^®^ laboratory blender (Seward). The liquid was removed by pipetting from the filter partition, transferred into a graduated 50-ml tube and the volume was determined. Then the tube was centrifuged at 10,000×*g* for 20 min at 4 °C (Heraeus Biofuge Stratos), the resulting supernatant was pipetted into a new 50-ml tube and 1.4 ml chloroform was added. After thoroughly mixing for 15 s, the tube was incubated at room temperature for 10 min, then centrifuged at 10,000×*g* for 15 min at 4 °C. The upper (aqueous) phase was transferred into a new tube and kept at 4 °C until RNA extraction.

To determine the efficiency of virus recovery from matrix samples, a process control sample was prepared by adding 50 µl of the MS2/MeV process control solution to a volume of PBS, equal to the volume of liquid recovered after sample homogenization (8–10 ml). The solution was kept at 4 °C until RNA extraction. For the HEV screening study of ready-to-eat meat products, one process control sample of an average volume of 9 ml was prepared for up to 12 matrix samples processed in parallel.

The extraction recovery rate of HEV was determined alike in spike experiments using 50 µl of diluted supernatant of cell culture infected with HEV genotype 3 strain 47832c (Johne et al. 2014). The amount of HEV corresponded to about 5 × 10^4^ genome copies. This HEV inoculum was added to previously HEV-negative tested matrix sample homogenates containing MS2/MeV process control solution, as well as to the corresponding volume of PBS (8-10 ml), also containing MS2/MeV process control solution. The solution was kept at 4 °C until RNA extraction.

### Determination of the Detection Limit

To determine the detection limit of HEV in matrix samples, cell culture supernatant infected with HEV genotype 3 strain 47832c (Johne et al. 2014) was serially diluted in PBS and an aliquot of 50 µl was added to HEV-negative matrix sample homogenates, together with 50 µl of the MS2/MeV process control solution. The virus extraction was performed as previously described.

### Isolation of Viral RNA

Total RNA was extracted from 1 ml of sample using the NucliSens^®^ magnetic extraction system (BioMérieux). Extraction was done manually with a magnetic rack, according to the user manual. RNA was eluted in 60 µl elution buffer and immediately used for further analysis or stored at − 80 °C.

### RT-qPCR for HEV, MS2 and MeV RNA Detection

Quantitative reverse transcription PCR (RT-qPCR) was performed on a Rotor-Gene Q thermocycler (Qiagen) in duplicate reactions. The QuantiTect Probe RT-PCR Kit (Qiagen) was used in a total reaction volume of 25 µl, with 5 µl RNA extract or PCR-grade water as no template control (NTC). Primer and probe concentrations were 0.4 and 0.2 µM, respectively. The oligonucleotide sequences are outlined in Table 1. Cycling conditions were as follows: 50 °C for 30 min, 95 °C for 15 min, then 45 cycles each with 94 °C for 10 s, 55 °C for 20 s and 72 °C for 60 s. Data acquisition was done after the extension step at 72 °C. For data analysis, the Rotor-Gene software version 2.2.3 was used. RNA samples that were tested positive for HEV (positive amplification signal for both duplicate reactions) were confirmed by a second RT-qPCR measurement. If duplicate reactions repeatedly resulted in a positive outcome, the sample was judged as HEV positive.

**Table 1:** Oligonucleotides used for RT-qPCR detection of HEV, MS2 and MeV RNA, and for genotyping of HEV

PCR system	Name	Sequence (5′–3′) and modifications	Amplicon length (bp)	References
HEV RT-qPCR	JVHEVF	GGTGGTTTCTGGGGTGAC	70	Jothikumar et al. (2006)
	JVHEVR	AGGGGTTGGTTGGATGAA		
	JVHEVP	FAM-TGATTCTCAGCCCTTCGC-BHQ1		
MS2 RT-qPCR	MS2-TM2-F	TGCTCGCGGATACCCG	61	Dreier et al. (2005)
	MS2-TM2-R	AACTTGCGTTCTCGAGCGAT		
	MS2-TM2JOE	JOE-ACCTCGGGTTTCCGTCTTGCTCGT-BHQ1		
MeV RT-qPCR	Mengo110	GCGGGTCCTGCCGAAAGT	100	Pinto et al. (2009)
	Mengo209	GAAGTAACATATAGACAGACGCACAC		
	Mengo147	Cy5-ATCACATTACTGGCCGAAGC-MGB NFQ		
HEV outer nested PCR	HEV-cs	TCGCGCATCACMTTYTTCCARAA	469–472	Johne et al. (2010)
	HEV-cas	GCCATGTTCCAGACDGTRTTCCA		
HEV inner nested PCR	HEV-csn	TGTGCTCTGTTTGGCCCNTGGTTYCG	331–334	
	HEV-casn	CCAGGCTCACCRGARTGYTTCTTCCA		

*FAM* 6-carboxyfluorescein, *BHQ1* black hole quencher 1, *JOE* 6-carboxy-4′,5′-dichloro-2′,7′-dimethoxyfluorescein, *Cy5* cyanine dye 5, *MGB* minor groove binder, *NFQ* non-fluorescent quencher, *bp* basepairs

### Calculation of Extraction Efficiency

The virus extraction efficiency or the recovery rate, expressed as a percentage (%), of the MS2 and MeV process controls or of HEV spiked to matrix samples, was calculated according to the equation:

$$E\text{ } = 2^{{ - \Delta {\text{Cq}}}} \times 100,$$

whereas ΔCq is the difference in the RT-qPCR quantification cycle (Cq) of the matrix sample and the corresponding process control sample. The Cq of the process control sample represented 100% extraction efficiency. Recovery rates of matrix samples slightly exceeding 100% due to variation of Cq values were rounded to 100%.

### Calculation of HEV Copy Numbers

The HEV genome copy number per gram of positive food samples was determined by HEV RT-qPCR in triplicate reactions, using a standard curve generated of a serially diluted HEV PCR product of known concentration. This HEV standard was previously amplified using extracted RNA (QIAamp Viral RNA Mini Kit, Qiagen) from supernatant of a cell culture infected with HEV genotype 3 strain 47832c (Johne et al. 2014). This RNA was further used as positive template control (PTC) in HEV RT-qPCR. The PCR product standard was purified (NucleoSpin^®^ Gel and PCR Clean-up Kit, Macherey–Nagel) and measured on a ND-1000 Spectrophotometer (NanoDrop). The copy number was calculated on the basis of the concentration in ng/µl and the molecular mass of the PCR product.

### Genotyping of HEV-Positive Samples

RNA extracts tested positive in the HEV RT-qPCR were further subjected to genotyping, which was performed following a published nested-PCR approach (Johne et al. 2010) with slight modifications. Synthesis of cDNA was done using the High-Capacity cDNA Reverse Transcription Kit (Applied Biosystems), in a total reaction volume of 20 µl with 10 µl RNA extract. Both outer and inner nested PCRs were done in a total reaction volume of 25 µl, with 5 µl cDNA for the outer nested PCR, and 5 µl reaction mix of the outer nested PCR for the inner nested PCR, respectively. One unit of Red Diamond Taq DNA polymerase (Eurogentec) was used with 1x reaction buffer, 2 mM MgCl_2_ and 0.2 mM dNTP each. Primer concentrations were 0.5 µM. The oligonucleotide sequences are outlined in Table 1. Cycling conditions were as follows: 95 °C for 3 min, then 40 cycles each with 94 °C for 30 s, 50 °C for 30 s and 72 °C for 60 s, with a final extension at 72 °C for 5 min. PCR products were separated on 1.5% agarose gel and visualized with HD Green Plus DNA stain (Intas) under UV-light. The inner nested PCR products were purified (NucleoSpin^®^ Gel and PCR Clean-up Kit, Macherey–Nagel) and DNA strands were sequenced (Microsynth) in separate forward- and reverse-primer reactions. The edited nucleotide sequences were analysed for phylogenetic relationship using the web service Phylogeny.fr (Dereeper et al. 2010, 2008). A set of previously described sequences with HEV genotypes 1–4 were used as reference (Johne et al. 2010).

## Results

### Optimizing of Virus Extraction

Our first attempts to establish a published method for HEV detection (Szabo et al. 2015) from 2-g samples of a liver sausage containing 18% pork liver resulted in low extraction recovery rates of the MS2 process control of 0.001% (Tables 2, 3). The recovery rate for 5-g samples of raw meat sausages was 0.4% (Table 3) which is still below 1%. An inhibitory effect of the liver sausage matrix on the virus and/or RNA extraction procedure and the RT-qPCR assay was suspected, but not further investigated in detail. Several measures for optimizing were tested. By adding 2 ml and 5 ml PBS buffer to the liver sausage samples in the homogenization step, the MS2 recovery rate increased from 0.001 to 0.01 and 0.2%, respectively (Table 2). Depending on the sample matrix, the volume of homogenate averaged 3–5 and 8–10 ml, for undiluted samples and samples with 5 ml PBS addition, respectively. Although the addition of 5 ml PBS increased the MS2 Cq value from 24.46 to 25.73, as was observed for the process control sample, the matrix sample MS2 Cq value decreased about more than 6, from 40.57 to 34.46 (Table 2). In spiking experiments where sample homogenates were artificially inoculated with HEV, it was shown that both MS2 and HEV recovery rates were concomitantly increased in a similar extent by the addition of PBS. A balance between overcoming the inhibitory effect and loss of analytical sensitivity due to sample dilution was achieved by adding 5 ml of PBS at most. The addition of PBS increased the recovery rates for MS2 process control for liver sausages and raw meat sausages from 0.001 to 0.1% and 0.4 to 2.2%, respectively (Table 3). For HEV, the recovery rates increased for both food matrices from 0.1 to 62.0% and 33.7 to 92.7%, respectively. Other diluents like sodium dodecyl sulphate (SDS) buffer commonly used for genomic DNA extraction of meat products [10 mM Tris–HCl (pH 8.0), 150 mM NaCl, 2 mM EDTA, 1% (w/v) SDS, 0.5 M guanidine-HCl] (Meyer et al. 1996) could not improve the recovery rates.

**Table 2:** Influence of PBS buffer on Cq values of MS2 and HEV RT-qPCR and virus extraction recovery rates measured for 2 g samples of liver sausage

	Undiluted (5 ml)			+2 ml PBS (7 ml)			+5 ml PBS (9 ml)		
	Cq _ms_	Cq _pcs_	RR (%)	Cq _ms_	Cq _pcs_	RR (%)	Cq _ms_	Cq _pcs_	RR (%)
MS2	40.57	24.46	0.001	37.70	24.94	0.01	34.46	25.73	0.2
HEV	37.78	31.20	1.0	34.81	31.88	13.1	31.54	31.96	100

The total lysate volume is indicated in brackets. Results were obtained from a single experiment

*Cq*_*ms*_ Cq value of matrix sample, *Cq*_*pcs*_ Cq value of process control sample, *RR* recovery rate

**Table 3:** Influence of PBS and SDS buffers on the MS2 and HEV virus extraction recovery rates of liver sausage and raw meat sausage

		Undiluted RR (%)	+5 ml PBS RR (%)	+5 ml SDS RR (%)
Liver sausage (2 g)	MS2	0.001	0.1	0.1
	HEV	0.1	62.0	49.7
Raw meat sausage (5 g)	MS2	0.4	2.2	2.1
	HEV	33.7	92.7	72.2

Results were obtained from a single experiment

*RR* recovery rate

The recovery rates observed for HEV were higher than for MS2. To test the influence of a particular process control virus on the extraction efficiency, MeV was evaluated as process control, in the same way that MS2. For both food matrices, the experiment revealed higher recovery rates for MeV (18.8, 51.4%), compared to MS2 (0.6, 27.6%), and HEV showing the highest values of 55.5 and 97.3%, respectively (Table 4). To assess the performance of both process controls on a broader database, MS2 as well as MeV were used for testing of ready-to-eat meat products containing pork liver or raw meat on the Swiss retail market.

**Table 4:** Virus extraction recovery rates of MS2 and MeV process controls and HEV, for liver sausage and raw meat sausage, with addition of 5 ml PBS buffer

	MS2 RR (%)	MeV RR (%)	HEV RR (%)
Liver sausage (2 g)	0.6	18.8	55.5
Raw meat sausage (5 g)	27.6	51.4	97.3

Results were obtained from a single experiment

*RR* recovery rate

The detection limit for HEV in matrix samples was about 1.56 x10^3^ and 1.56 x10^2^ genome copies per gram of liver sausage and raw meat sausage, respectively (Table 5). Over the whole HEV dilution range high recovery rates between 10.4 and 33.0% were measured.

**Table 5:** Detection limit of HEV in matrix samples, the corresponding virus extraction recovery rates of MS2 and MeV process controls and HEV, and the Cq values of HEV RT-qPCR

		HEV inoculation level [log_10_ genome copies]					
		4.70	4.10	3.49	2.89	2.29	NTC
Liver sausage (2 g)	MS2 RR (%)	15.3	9.4	7.3	5.0	25.5	16.9
	MeV RR (%)	39.0	31.5	29.0	21.6	33.8	60.7
	HEV RR (%)	24.0	28.1	26.8	–	–	–
	HEV Cq _ms_ HEV Cq _pcs_	32.70 30.64	35.21 33.38	38.26 36.36	– 38.37	– –	– –
Raw meat sausage (5 g)	MS2 RR (%)	0.3	1.1	2.2	3.1	0.9	0.1
	MeV RR (%)	19.3	35.4	43.4	32.1	11.5	6.8
	HEV RR (%)	14.8	28.1	33.0	10.4	–	–
	HEV Cq _ms_ HEV Cq _pcs_	33.56 30.80	34.97 33.14	37.34 35.74	39.77 36.51	– –	– –

Results were obtained from a single experiment

*RR* recovery rate, *Cq*_*ms*_ Cq value of matrix sample, *Cq*_*pcs*_ Cq value of process control sample, *NTC* no template control (matrix sample without HEV inoculation)

### Screening of Ready-to-Eat Meat Products from the Swiss Retail Market

A total of 90 samples of pork liver sausages (37) and raw meat sausages (53) were analysed for HEV using the previously optimized method (Tables 6, 7). HEV-RNA was detected in 10 samples (11.1%), 7 of which were liver sausages and 3 raw meat sausages. This represents a HEV-positive rate of 18.9% for liver sausages and 5.7% for raw meat sausages. Three of the positive samples were imported products from Germany. 22 of the 53 analysed raw meat sausages (13 “Mortadella cruda” and 9 “Lebersalsiz”) represented high-risk products to be contaminated with infectious HEV particles since they contained raw pork liver. HEV-RNA was detected in 3 of these samples (13.6%), twice “Mortadella cruda” and once “Lebersalsiz”. No HEV-RNA was detected in the other 31 raw meat sausages that did not contain liver, including 15 products made of game meat like deer (7), wild boar (5), chamois (2) and ibex (1). The range of virus load was between 1.72 and 5.54 log_10_ HEV genome copies per gram (Table 7).

**Table 6:** Results of the HEV screening of meat products from the Swiss retail market

	Samples tested	HEV-positive samples	HEV-positive rate (%)
Liver sausages	37	7	18.9
Raw meat sausages	53	3	5.7
With raw liver	22	3	13.6
With game meat	15	0	0
Total	90	10	11.1

**Table 7:** Product type and origin, virus extraction recovery rates of the MS2 and MeV process controls, mean Cq value of HEV RT-qPCR, and genotype of HEV-positive samples

Product type	Country of origin	MS2 RR (%)	MeV RR (%)	HEV RT-qPCR (Cq value)	HEV (log_10_ genome copies/g)	HEV genotype
Liver sausage	CH	0.2	n.d.	31.97	4.01	3
Liver sausage	CH	6.1	n.d.	36.86	2.51	n.d.
Liver sausage	CH	2.8	n.d.	28.38	5.10	3
Liver sausage	DE	1.2	25.3	26.97	5.54	3
Liver sausage	DE	1.3	25.8	31.19	4.25	3
Liver sausage	DE	1.6	11.5	29.97	4.62	3
Liver sausage	CH	3.2	34.3	34.42	3.26	3
Raw meat sausage^a^	CH	0.01	0.2	38.17	1.72	3
Raw meat sausage^b^	CH	0.01	0.7	32.35	3.49	3
Raw meat sausage^b^	CH	0.5	6.0	34.34	2.89	3

*RR* recovery rate, *CH* Switzerland, *DE* Germany, *n.d.* not determined

^a^Local sausage specialty “Lebesalsiz”

^b^Local sausage specialty “Mortadella cruda”

For all samples, the determined average virus extraction recovery rates for the MS2 and MeV process controls were 10.0 and 24.4%, respectively (Table 8).

**Table 8:** Virus extraction recovery rates (mean, min, max, and median values) of MS2 and MeV process controls for different sample subsets

	*N*	MS2 RR (%)				MeV RR (%)			
		Mean	Min	Max	Median	Mean	Min	Max	Median
All samples	90	10.0	0.01	100	1.2	24.4	0.1	100	14.3
Liver sausage	37	22.3	0.01	100	3.2	41.2	0.8	100	32.4
Raw meat sausage	53	1.4	0.01	15.6	0.4	12.0	0.1	57.4	4.7
HEV-positive samples	10	1.7	0.01	6.1	1.3	14.8	0.2	34.3	11.5

*n* number of samples, *RR* recovery rate

### Genotyping of HEV-Positive Samples

Nine of the 10 HEV-positive tested samples were successfully genotyped. The mean Cq values of the HEV RT-qPCR ranged from 26.97 to 38.17. In one sample of liver sausage with a Cq value of 36.86 genotyping was not successful. The analysis of the phylogenetic relationship of the inner nested PCR product sequences revealed an unambiguous assignment to HEV genotype 3 (Table 7).

## Discussion

### Optimizing of Virus Extraction

For reliable detection and quantification of viruses in food samples, a method featuring a high extraction efficiency is vital. The optimization of such methods is therefore crucial to achieve a low detection limit and for accurate measurement of the actual virus load. In a recently conducted study (Szabo et al. 2015), different published extraction methods (Baert et al. 2008; Stals et al. 2011) were assessed for their recovery rates by testing of a raw sausage matrix using MS2 as process control virus, as a surrogate for HEV. The range of recovery rates was between 0.04 and 1.92%, whereas for the same sample matrix inoculated with HEV a recovery rate of 4.9% was achieved. Their Trizol^®^-based protocol was further optimized with regard to the homogenization technique. Thus, the mean extraction efficiency of MS2 for raw sausages raised to 11.2%, but for liver sausages it did not exceed 1%. Based on this experience, we addressed the issue of improving the virus recovery rate in our study, especially for difficult food matrices like liver sausage. The addition of PBS buffer to the sample in the homogenization step resulted in an increased extraction efficiency for MS2. Even higher recovery rates were obtained by using MeV as process control instead of MS2. For liver sausages, the range of recovery rates for MeV was from 0.8 to 100%, with a mean 41.2%, showing a high variability in recovery rates (Table 8). The range of recovery rates for HEV was between 10.4 and 100% (Tables 2, 5) for both inoculated sample matrices.

If required, our method can easily be scaled up for homogenization of higher amounts of sample material, just by keeping the proportion of sample and liquids, e.g. PBS and TRI Reagent^®^ Solution. The procedure of virus extraction is simple and can be done with standard equipment and consumables that are available in a microbiological laboratory. Another method for HEV extraction of increased sample size of 25-g pork meat products has been published recently (Mykytczuk et al. 2017). They reached an average extraction efficiency of 5.45% for the feline calicivirus (FCV) process control and 5.63% for HAV, but their method is laborious, time-consuming and uses an in-house constructed filter device.

The detection limit for HEV in liver sausages and raw meat sausages was determined to be 5.34 × 10^4^ and 2.93 × 10^3^ genome equivalents per gram, respectively (Szabo et al. 2015). Similar results were obtained in a previous study investigating swine organs and tissues at slaughterhouse (Leblanc et al. 2010). The limit of detection of our optimized method was tenfold lower, about 1.56 × 10^3^ and 1.56 × 10^2^ genome copies per gram, for liver sausages and raw meat sausages, respectively (Table 5). A higher sensitivity could for example be achieved by HEV replication prior to RT-qPCR detection, e.g. in a cell culture-based in vitro assay or by other alternative techniques.

### Screening of Ready-to-Eat Meat Products

Since no data are available on the frequency of contamination and viral loads of HEV in meat products from the Swiss retail market, this study aimed to close this gap at least for ready-to-eat products containing pork liver or raw meat, which are suspected to be a source for HEV infections of humans. Our findings for the prevalence of HEV in liver sausages (18.9%) and raw meat sausages (5.7%) revealed similar results as presented in other studies conducted in Europe, in which positive rates of 4–31% were reported (Berto et al. 2012; Di Bartolo et al. 2012, 2017; Martin-Latil et al. 2014; Szabo et al. 2015; Pavio et al. 2014). Based on HEV prevalence in pork livers, a study assessing the risk of food-borne transmission of hepatitis E in Switzerland estimated that < 6% of the products containing pork liver and < 3% of the products containing pork could be contaminated with HEV genomic material (Muller et al. 2017). However, our experimental data revealed a higher prevalence. Regarding the viral load in pork products at retail, their estimates ranged between 1.6 and 3.5 log_10_ HEV genome copies per gram. Another study reported a similar value of 3.7 log_10_ viral units per gram for the estimated prevalence of HEV-RNA in porcine-derived food in Switzerland and Germany (Sarno et al. 2017). Our experiments have given slightly higher values between 1.72 and 5.54 log_10_ HEV genome copies per gram. In other studies, similar results for HEV loads of up to 6 log_10_ genome copies per gram were measured for dried and fresh liver sausages (Colson et al. 2010; Pavio et al. 2014). These data may even underestimate the true HEV load in such products since they have been collected with virus extraction methods showing lower recovery rates.

Interestingly we did not find HEV in raw meat sausages made of game meat like wild boar or deer, even though positive rates of 10% were reported for wild boar sausages in Germany (Szabo et al. 2015) and high seroprevalence rates of 12.5 and 29.9–41.3% were measured for Switzerland and Germany, respectively (Adlhoch et al. 2009; Burri et al. 2014; Schielke et al. 2015).

All sequences retrieved from HEV-positive samples belonged to HEV genotype 3, which further supports the conclusive presumption that pigs are a reservoir of this zoonotic genotype. The sample for which genotyping failed, a rather high Cq value of 36.86 was measured. Since outer and inner nested PCR products of the HEV genotyping approach are longer than the HEV RT-qPCR product (about 470 bp and 330 bp versus 70 bp), it is possible that for strongly processed matrices with reduced RNA integrity the amplification of larger PCR templates will fail.

### Public Health Considerations

The HEV detection method developed in this study was applied to test certain categories of meat products for HEV, to find out frequencies of contamination, to measure viral loads and to genotype isolated HEV sequences. The obtained data showed us that HEV occurs rather frequently in certain pork meat and pork liver products and that in some samples high HEV-RNA loads of up to 5.54 log_10_ genome copies per gram can occur. Our findings have to be seen as elements for future risk-assessments. For a full and complete risk-assessment, much more information would be needed. For example, it would be necessary to exactly know the production processes of analysed foods. Since we tested products purchased at retail level, this information was not available. There are also gaps of knowledge concerning the survival of HEV in food matrices such as ready-to-eat raw meat products containing pork or pork liver. Furthermore, there is a lack of information with regard to the infectious dose of HEV or the dose–response relationship, respectively (Sarno et al. 2017). To clarify that particular question, attempts of cases-source-attributions might be helpful (Muller et al. 2017). However, precondition for such studies would be the mandatory reporting of human hepatitis E infections what is currently not the case in Switzerland. Future studies should also try to quantitatively differentiate between inactivated and infectious HEV particles in contaminated foods which would imply the availability of appropriate cell culture systems. Although there are still several questions to answer, the data in this study and already published epidemiological findings justify certain precautionary measures. For the time being, our recommendation is that raw pork liver has to be thoroughly heated prior to consumption and that the fabrication processes of meat products containing pork liver must contain a virus-inactivating hurdle.
